# HIV status disclosure to male partners among rural Nigerian women along the prevention of mother-to-child transmission of HIV cascade: a mixed methods study

**DOI:** 10.1186/s12978-018-0474-y

**Published:** 2018-03-02

**Authors:** Angela Odiachi, Salome Erekaha, Llewellyn J. Cornelius, Christopher Isah, Habib O. Ramadhani, Laura Rapoport, Nadia A. Sam-Agudu

**Affiliations:** 1 Abuja, Nigeria; 2 International Research Center of Excellence, Institute of Human Virology Nigeria, Abuja, Nigeria; 3 School of Social Work and College of Public Health, University of Georgia Athens, Athens, USA; 4 Institute of Human Virology, University of Maryland School of Medicine, Baltimore, USA; 5 Harvard T. H. Chan School of Public Health, Boston, USA

**Keywords:** HIV, Disclosure, PMTCT, Serodiscordance, Male partner, Nigeria

## Abstract

**Background:**

HIV status disclosure to male partners is important for optimal outcomes in the prevention of mother-to-child transmission of HIV (PMTCT). Depending on timing of HIV diagnosis or pregnancy status, readiness to disclose and disclosure rates may differ among HIV-positive women. We sought to determine rates, patterns, and experiences of disclosure among Nigerian women along the PMTCT cascade.

**Methods:**

HIV-positive women in rural North-Central Nigeria were purposively recruited according to their PMTCT cascade status: pregnant-newly HIV-diagnosed, pregnant-in care, postpartum, and lost-to-follow-up (LTFU). Participants were surveyed to determine rates of disclosure to male partners and others; in-depth interviews evaluated disclosure patterns and experiences. Tests of association were applied to quantitative data. Qualitative data were manually analysed by theme and content using the constant comparative method in a Grounded Theory approach.

**Results:**

We interviewed 100 women; 69% were 21–30 years old, and 86% were married. There were 25, 26, 28 and 21 women in the newly-diagnosed, in-care, postpartum, and LTFU groups, respectively. Approximately 81% of all participants reported disclosing to anyone; however, family members were typically disclosed to first. Ultimately, more women had disclosed to male partners (85%) than to family members (55%). Rates of disclosure to anyone varied between groups: newly-diagnosed and LTFU women had the lowest (56%) and highest (100%) rates, respectively (*p* = 0.001). However, family (*p* = 0.402) and male partner (*p* = 0.218) disclosure rates were similar between cascade groups. Across all cascade groups, fear of divorce and intimate partner violence deterred women from disclosing to male partners. However, participants reported that with assistance from healthcare workers, disclosure and post-disclosure experiences were mostly positive.

**Conclusion:**

In our study cohort, although disclosure to male partners was overall higher, family members appeared more approachable for initial disclosure. Across cascade groups, male partners were ultimately disclosed to at rates > 75%, with no significant inter-group differences. Fear appears to be a major reason for non-disclosure or delayed disclosure by women to male partners. Augmentation of healthcare workers’ skills and involvement can mediate gender power differentials, minimize fear and shorten time to male partner disclosure among women living with HIV, regardless of their PMTCT cascade status.

**Trial registration:**

Clinicaltrials.gov registration number NCT 01936753, September 3, 2013 (retrospectively registered).

## Plain English summary

For women living with HIV, disclosure to male partners is important in preventing HIV transmission to infants and staying healthy on treatment. Gender inequality plays a key role in low rates of disclosure by women to male partners. In addition, HIV disclosure rates may differ depending on whether the woman was recently or previously diagnosed, or whether she is pregnant or has delivered. We interviewed 100 women living with HIV in rural North-Central Nigeria to evaluate their disclosure history and experiences. The women were pregnant and newly or previously HIV-diagnosed, breastfeeding, or had dropped out of HIV care.

Most women (81%) reported disclosing to anyone; with more disclosing to male partners than relatives (85% versus 55%). However, family members were typically disclosed to, first. Also, newly-diagnosed and out-of-HIV-care women were least and most likely, respectively, to disclose to anyone. Male partner disclosure rates were similar across groups. Women who disclosed to male partners did so to motivate them to test for HIV and to keep open, honest couples’ communication. Women across all groups reported avoiding male partner disclosure due to fear of divorce and violence. However, when healthcare workers were involved, disclosure experiences were mostly positive.

Our results show that family members were more approachable than male partners for initial disclosure, and that healthcare workers can, and have been instrumental in improving male partner disclosure experiences among HIV-positive women. Therefore, healthcare workers should be trained and proactively involved in helping HIV-positive women to disclose to male partners.

## Background

In 2016, there were an estimated 3.2 million people living with HIV in Nigeria, at a prevalence rate of 2.9% in the general population [1]. Unprotected heterosexual sexual intercourse remains the main mode of HIV transmission in Nigeria [2]. Latest available data show HIV prevalence at 3.5% in adult females versus 3.3% in males; and 3.6% and 3.2% in rural versus urban areas, respectively [2]. In 2016, only 34% of Nigeria’s large HIV-positive population were estimated to know their HIV status, and only 30% of those diagnosed received antiretroviral therapy (ART) [1]. Linkage to treatment is important for HIV prevention, because once an HIV-positive client is initiated and is compliant on a suppressive ART regimen, the risk of onward transmission drops significantly [3]. Thus, knowledge of HIV status through massive scale-up of HIV testing, and subsequent linkage to suppressive treatment is critical to containing the HIV epidemic.

Besides personal knowledge of HIV status, disclosure of such status by people living with HIV to others – family, friends, sexual partners – is important for HIV prevention, including the prevention of mother-to-child transmission of HIV (PMTCT) [4]. Disclosure facilitates treatment uptake, drug adherence and retention in care for people living with HIV, including pregnant women [4–10]. For this population, disclosure is important for dual prevention of HIV transmission to sexual partners and for PMTCT, through promoting male partner HIV testing, the adoption of safer sex practices, and partner support for PMTCT service uptake [11, 12].

The PMTCT cascade is a multistep continuum of care package to be completed by HIV-positive mother-exposed infant pairs, and includes maternal HIV testing and treatment, antenatal and delivery care, early infant diagnosis, postnatal services, and linkage to long-term HIV care and support [13]. Women who have disclosed have higher rates of antenatal care (ANC) uptake, facility delivery, and PMTCT ART use compared to women who have not [7]. Non-disclosure has been reported as a predictor of PMTCT cascade dropout [14], while women who disclosed to their partners were up to five times more likely to access and be retained in PMTCT care [15].

Despite the benefits, disclosure rates in PMTCT are widely disparate, particularly in sub-Saharan Africa. Among pregnant and post-partum African women living with HIV, disclosure rates to any person range between 5% and 97% (pooled estimate 67%), and to male partners, 30 to 93% (pooled estimate 64%) [16]. Gender inequities often rooted in socio-cultural factors play a key role in low rates of HIV status disclosure to male partners among women living with HIV [17]. Reasons for male partner non-disclosure among these women include fear of abandonment with the resultant loss of emotional, material and financial support [17–22]; emotional abuse, including name-calling, accusations of infidelity and exposing the family to HIV [19, 20, 22], and sex deprivation [23, 24]. Stigma as well as perceived or enacted discrimination - from male partners and/or the community at large-have also been reported [19, 25]. In extreme cases, women do not disclose for fear of physical violence and other forms of intimate partner violence [17, 19, 26, 27].

As a result of HIV testing in pregnancy, women are often diagnosed before their male partners-regardless of who was infected first-and assume the added burden and responsibility of disclosure [25, 26]. Such gendered asymmetrical disclosure – where only one partner discloses - affects women disproportionately and negatively, as the partner who tests positive first is considered the unfaithful partner and “cause” of the infection, even though the male sexual partner may already be infected [22, 26]. Among HIV-positive women, the first choice of whom to disclose to is often not their male sexual partner(s); rather, where disclosure occurs, it is usually first to a trusted family member who is expected to provide social support [21]. Disclosure to a male partner may occur later or not at all [28, 29]. Despite pre-disclosure fears, many women who disclose report surprisingly positive reactions and support from family and male partners [6, 12, 15, 17, 21, 22, 30, 31]. Nonetheless, negative consequences have also been reported [6, 12, 15, 17, 18, 21, 26, 27].

The reasons for (non)-disclosure and therefore disclosure rates may differ depending on where a woman may be in her PMTCT or HIV treatment journey. Studies in Nigeria have reported HIV disclosure rates from women to male partners between 23.0% and 75.6% in often urban ART clinics [24, 32–34]; and 90.4% among pregnant women [27]. However there is little differentiated data on rates of disclosure among women at different points along the PMTCT cascade, particularly in programmatically challenging rural areas.

Nigeria is an especially important target for scale-up of impactful strategies in maternal and child health and PMTCT. The country has large gaps, especially in rural settings, including low rates of skilled ANC uptake in rural (46.5%) vs urban (86.0%) areas; and facility delivery for only 21.9% of rural, versus 61.7% of urban women [35]. PMTCT gaps include low maternal ART coverage and poor early infant diagnosis uptake of only 30% and 9%, respectively [36]. Studies discussed above highlight the need for evidence to inform robust socio-behavioral interventions targeting key issues like non-disclosure, to augment biomedical PMTCT strategies in Nigeria and similar settings. This study sought to determine the rates, patterns and experiences of disclosure, primarily to male partners, among women at different stages of the PMTCT cascade in rural Nigeria.

## Methods

### Study design and setting

This cross-sectional, concurrent mixed-methods study was conducted between July and November 2013, and was nested in the MoMent Nigeria PMTCT implementation research project [37]. The study reported here was designed to understand the rates and context of disclosure (or lack thereof) among women living with HIV. The focus on differences along the PMTCT cascade prompted the study to target core PMTCT consumers (women), and not their male partners. The study was conducted in two high HIV-burden states of Nasarawa and the Federal Capital Territory, with 8.1% and 7.5% general population seroprevalence rates, respectively [2]. Both study states are contiguously located in North-Central Nigeria. The study sites comprised 14 primary healthcare centers and two secondary-level facilities located in rural communities participating in the prospective MoMent study [37]. At the time of the study, all sites were implementing World Health Organization (WHO) Option B regimens per national guidelines, including initiation of maternal ART regardless of CD4 count at booking, and infant breastfeeding concurrent with maternal ART [38].

### Study participants and recruitment

Eligible women were HIV-positive, ≥ 18 years old, who were receiving or had previously received PMTCT services at the study sites. Participants were recruited in four groups according to their position along the PMTCT cascade at the time of the study:

- Pregnant, newly HIV-diagnosed (within 7 days), not yet on ART *(“newly-diagnosed women*”)
- Pregnant, on ART, in PMTCT care (“*ANC women”*)
- Post-partum (up to three months), breastfeeding, on ART, in care (“*postpartum women*”)
- Previously in PMTCT care, lost to follow-up (LTFU), not on ART (“*LTFU women*”). LTFU women were defined as those who had not completed a facility visit in three or more consecutive months.

Women who had not been formally enrolled in PMTCT care and did not have medical records at the recruiting study sites were excluded. We targeted a sample size of 100 participants along the PMTCT cascade, based on recruitment capacity estimations derived from enrollments for women living with HIV at the study sites.

Healthcare workers at study sites identified eligible women during routine clinic visits and contacted LTFU women identified from facility service registers by phone. Thereafter, all interested women were approached by study staff for written informed consent. Self-reported HIV status from participants was crosschecked with medical records at each study facility. To ensure that recruitment calls did not put women at risk of confidentiality breaches, all recruitment calls were made by healthcare workers who only provided details about the call if it was answered by the verified potential study participant; otherwise, a cryptic message or excuse was given by the caller. Those who were not reached by phone, especially those LTFU, were tracked with the assistance of Mentor Mothers (women living with HIV serving as peer counselors at study facilities).

### Data collection and analyses

A three-section semi-structured interview guide was used to simultaneously collect quantitative and qualitative data. The first section of the guide collected information on participant socio-demographics (including age, religion, marital status and parity). The second section collected disclosure data (including whether patient had disclosed their status, to whom, and in what order), and data on knowledge of male partner HIV status. The third section collected qualitative information that explored each participant’s “lived experience” with disclosure as a woman living with HIV. The key question posed to participants was, “Is there anyone who knows you have HIV?” This was followed by other questions to determine the process, and reasons for disclosure or non-disclosure. The guide was pilot-tested among 10 women and then updated and finalized before implementation.

Two trained study staff fluent in English and the dominant Hausa local language conducted each face-to-face interview in either language, using the semi-structured interview guide. While one study staff interviewed, the other observed and took notes. All interviews were audio-taped, and took place in private rooms at study sites or other designated locations by participant request. Each interview lasted 45 min to one hour. Healthcare workers at study sites neither participated in, nor observed the study sessions. Both English and Hausa audio-taped interviews were transcribed (and where relevant, translated) verbatim in English.

#### Quantitative data analysis

Participants’ socio-demographic and disclosure data (including disclosure status and knowledge of male partner HIV status) were first analysed with descriptive statistics. This was followed by tests for associations between the independent categorical variable “PMTCT cascade group,” and dependent categorical variables, including “disclosure to male partners/others” and “knowledge of partner HIV status” using Fisher’s Exact test. Statistical Package for Social Sciences version 16.0 for Windows was used for analysis, and statistical significance was set at *p* ≤ 0.05.

#### Qualitative data analysis

All interview transcripts and field notes were analysed manually by theme and content using the constant comparative method in a Grounded Theory approach [39]. In this approach, inductive methodology is used to systematically generate theory from the data collected. Qualitative analysis was performed by a panel of eight trained and/or experienced researchers including SE, CI, LR, NASA, and an experienced Social Scientist (LJC). We selected a series of code words-the initial code word being “disclosure”-to develop themes and sub-themes from the qualitative data. This led to an iterative content analysis of the transcripts to examine the overall conceptual issues that emerged.

During this process, each researcher independently used the code list to hand-code assigned transcripts by reviewing and summarizing each line, phrase and paragraph to identify key themes. This was followed by group review, triangulation, and content analysis by iteration until a final consensus on patterns and categorizations was achieved. The research team that facilitated the in-depth interviews was maintained for completing transcription as well as conducting the qualitative analysis. AO additionally independently analyzed the transcripts and coded data based on identified themes from the interview guide, and compared these to themes identified by the paired researchers.

## Results

A total of 100 women were recruited in the four targeted PMTCT cascade groups: 25 newly-diagnosed, 26 in ANC, 28 postpartum, and 21 LTFU women (Table 1). Overall, 69% (69/100) study participants were between 21 and 30 years old, and 88.0% (88/100) had at least primary school education. The majority (86%, 86/100) of participants were married. Recruited participants had similar characteristics across all four cascade groups except for marital status: women in the LTFU group were more likely to be single, compared to the other 3 groups (Table 1). We collected data for both quantitative and qualitative analyses from all 100 women.

**Table 1:** Respondents’ socio-demographic characteristics

Characteristic	Newly- diagnosed	ANC	Postpartum	Lost to follow-up	*P* value*	Total
	*N* = 25	*N* = 26	*N* = 28	*N* = 21		*N* = 100
	n (%)	n (%)	n (%)	n (%)		n (%)
Age, years						
< 21	4 (16.0)	0 (0.0)	0 (0.0)	3 (13.6)	0.327	7 (7.0)
21–30	18 (72.0)	16 (61.5)	22 (78.6)	13 (59.1)		69 (69.0)
31–40	3 (12.0)	10 (38.5)	6 (21.4)	5 (27.3)		24 (24.0)
Religious affiliation						
Christian	16 (66.7)	18 (69.2)	25 (92.6)	16 (76.2)	1.000	75 (76.5)
Muslim	8 (33.0)	8 (30.8)	2 (7.4)	5 (23.8)		23 (23.5)
No response	1	0	1	0		2
Marital status						
Single^a^	0 (0.0)	3 (11.5)	3 (10.7)	8 (38.1)	0.001	14 (14.0)
Married	25 (100.0)	23 (88.5)	25 (89.3)	13 (61.9)		86 (86.0)
Number of living children						
None	7 (28.0)	7 (27.0)	1 (3.7)	4 (20.0)	0.058	19 (19.5)
1–2	9 (36.0)	13 (50.0)	19 (70.4)	5 (25.0)		46 (46.9)
3–4	7 (28.0)	5 (19.2)	6 (22.2)	8 (40.0)		26 (26.5)
≥ 5	2 (8.0)	1 (3.8)	1 (3.7)	3 (15.0)		7 (7.1)
No response	0	0	1	1		2

Newly-diagnosed: women pregnant and newly HIV-diagnosed within last 7 days

ANC: women pregnant and in antenatal care

Postpartum: Breastfeeding women within 3 months of delivery

Lost-to follow-up: women who had not attended a clinic visit in 3 or more consecutive months

*Fisher’s Exact test

^a^Includes single, widowed and divorced women

### Results from quantitative data analysis

#### Rates of HIV status disclosure among participants

Participants were asked about disclosure of their HIV-positive serostatus to male partners or first-order family members (parents and/or siblings). Approximately 81% of participants reported disclosing to anyone; of these, more had disclosed to their male partner than to family members (85.0% vs 54.5%) (Table 2). At 100%, LTFU women had the highest rates of disclosure to anyone. Newly-diagnosed women had the lowest disclosure rates to anyone or family, while postpartum women had the lowest disclosure rates to male partners. Interestingly, while newly-diagnosed women had the lowest disclosure rates to anyone, they had comparable or higher male partner disclosure rates compared to the other cascade groups. Analysis showed significantly different disclosure rates to anyone across the four groups (*p* = 0.001); however there were no significant differences in disclosure rates to family members (*p* = 0.402) or male partners (*p* = 0.218) (Table 2). Similarly, when compared across pregnant (newly-diagnosed + ANC) and non-pregnant (postpartum + LTFU) women, disclosure rates to anyone were lower among pregnant women (56% for newly-diagnosed women and 84% among ANC women), and were significantly different (*p* = 0.0007) compared to non-pregnant women; but not for disclosure to family (*p* = 0.653) or male partners (*p* = 1.000).

**Table 2:** Disclosure by women living with HIV along the PMTCT cascade

Disclosure status	Newly-diagnosed	ANC	Postpartum	Lost to follow-up	Total	*P* value^a^
	*N* = 25	*N* = 26	*N* = 28	*N* = 21	*N* = 100	
	n (%)	n (%)	n (%)	n (%)	n (%)	
Disclosed to anyone						
Yes	14 (56.0)	21 (84.0)	24 (85.7)	21 (100.0)	80 (80.8)	0.001
No	11 (44.0)	4 (16.0)	4 (14.3)	0 (0.0)	19	
No response	0	1	0	0	(19.2) 1	
Disclosed to family^b^	*N* = 14	*N* = 21	*N* = 24	*N* = 21	*N* = 80	
Yes	5 (35.7)	12 (63.2)	12 (52.2)	13 (61.9)	42 (54.5)	0.402
No	9 (64.3)	7 (36.8)	11 (47.8)	8 (38.1)	35	
No response	0	2	1	0	(45.5) 3	
Disclosed to male partner^b^	*N* = 14	*N* = 21	*N* = 24	*N* = 21	*N* = 80	
Yes	13 (92.9)	17 (81.0)	18 (75.0)	20 (95.2)	68 (85.0)	0.218
No	1 (7.1)	4 (19.0)	6 (25.0)	1 (4.8)	12 (15.0)	
No response	0	0	0	0	0	

Newly-diagnosed: women pregnant and newly HIV-diagnosed within last 7 days

ANC: women pregnant and in antenatal care

Postpartum: Breastfeeding women within 3 months of delivery

Lost-to follow-up: women who had not attended a clinic visit in 3 or more consecutive months

^a^Fisher’s exact test

^b^Participants responding “No” to “Disclosed to anyone” have been removed from denominator

#### Participants’ knowledge of male partners’ HIV status

Approximately 67% (54/81) of respondents knew their partner’s HIV status, while one-third did not (Table 3). There were significant differences in knowledge of male partner’s HIV status across the four groups (*p* = 0.004). The newly-diagnosed group had the lowest proportion of women who knew their partner’s status, while the LTFU group had the highest. Among 54 women who knew their partner’s status, 30 reported he was HIV-negative, indicating an overall serodiscordance rate of 56%.

**Table 3:** Knowledge of Male Partner’s HIV status among Women Living with HIV

Partner’s HIV Status	Newly-diagnosed	ANC	Postpartum	Lost to follow-up	Total	*P* value^a^
	*N* = 25	*N* = 26	*N* = 28	*N* = 21	*N* = 100	
	n (%)	n (%)	n (%)	n (%)	n (%)	
Positive	4 (19.1)	8 (38.1)	5 (21.7)	7 (43.8)	24 (29.6)	
Negative	2 (9.5)	7 (33.3)	14 (60.9)	7 (43.8)	30 (37.0)	
Unknown	15 (71.4)	6 (28.6)	4 (17.4)	2 (12.4)	27 (33.4)	0.004
No response	4	5	5	5	19	

Newly-diagnosed: women pregnant and newly HIV-diagnosed within last 7 days

ANC: women pregnant and in antenatal care

Postpartum: Breastfeeding women within 3 months of delivery

Lost-to follow-up: women who had not attended a clinic visit in 3 or more consecutive months

^a^Fisher’s Exact test

### Results from qualitative data analysis

**Fig. 1:**
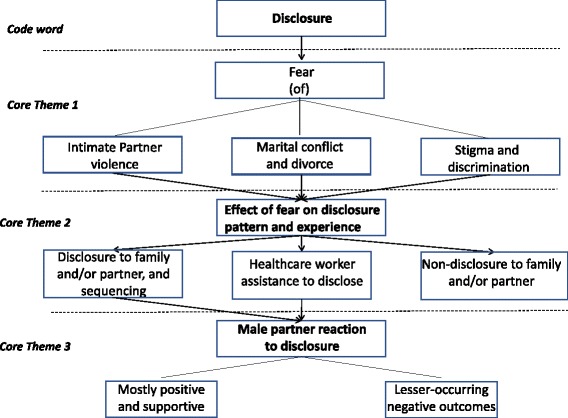
Core emerging themes from qualitative data analysis

Figure 1 displays the core themes that emerged from qualitative data analysis.

#### Effect of fear on disclosure pattern and experience

One consistent theme that emerged in the qualitative analysis was descriptions of worry and stress experienced by participants, either as they considered disclosing, or as a result of disclosure, particularly with respect to male partners. Digging deeper into the data, we found that fear of marital conflict – in the form of intimate partner violence, or divorce – were important factors during the disclosure process across all four groups:: “*I have not even told my husband, because I don’t want to lose my marriage. He is a very difficult person”*- Newly-diagnosed woman. *“Of course he will divorce me since the disease is a license to death”-*Newly-diagnosed woman*.* “*I feared that telling him would cause a fight”-*LTFU woman*. “I am scared of him beating me and divorcing me at the end”-*Postpartum woman. Some women mentioned being afraid, but failed to explain why, even after probing: *“No, I’m just afraid; I do not know how to tell him”-*Postpartum woman*. “I just feel like not telling him… I know I will tell him, but not now…Yes, he needs to know but it is something you take gradually*”-Postpartum woman.

Nearly 20% of study participants had not disclosed to anyone (Table 2), among whom were women who expressed no intention to disclose their status to male partners and/or others, largely due to uncertainty about the nature of ensuing reactions and/or stigma. *“I haven’t told anyone because nobody in my family has experienced anything like this and this is a big shock to me, hearing that I have HIV”-*ANC woman. “*If I tell* [anyone], *I do not know what the outcome will be”-*Newly-diagnosed woman. *“Some people believe that HIV is only from promiscuous people”-*ANC woman*.*

The fear of marital conflict influenced not only whether women disclosed at all, or whom they disclosed to, but also the timing and pattern of disclosure. While most women who disclosed, disclosed immediately or within days to others, they found it most difficult to disclose to male partners. One respondent stated: “*Only my sister knows. When they told me then I was sad. So I just went straight to her house to tell her…When I told her she was equally sad… I didn’t tell him* [husband]”-ANC woman, partner status unknown. “*I called her* [sister] *after I have been told about the diagnosis in the hospital*. *She later found time to talk with me and encourage me about living with it*…[But] *he* [husband] *is not aware yet*” -Postpartum woman. *“I spoke with her* [friend] *after getting my diagnosis from the hospital. I explained everything to her since I cannot tell my husband. But truly, I am afraid of telling him because it is not something that is easy to disclose” -* Postpartum woman, partner status unknown.

Based on this fear, some women disclosed to only family, and not partners. However, women who first disclosed to male partners were sometimes asked by the latter to refrain from disclosing to anyone else, including family members: *“He begged me not to tell even my family; that we should keep it a secret between the two of us”* -ANC woman, partner HIV-negative. Such partners’ requests may be as a result of pride, or fear of stigma “by association,” prompting the men to want to protect their families, and their integrity and status in the home and/or community.

Our quantitative analysis showed that nearly half (42%) of newly-diagnosed women had not disclosed to anyone (Table 2), but most stated that they planned to disclose to their male partners: *“I plan to tell my husband very soon*.” “*I will tell him when I reach home.”* Given the immediacy of the HIV-positive diagnosis (seven days or less), there was less data from newly-diagnosed women on disclosure and post-disclosure experiences, especially involving male partners.

Besides fear, some women did not disclose to their male partner due to mistrust, anger and suspicion that he was responsible for their HIV-positive status through infidelity: “[I have not told him] *because I think that he is the one that gave HIV to me, because he doesn’t stay at home”-* Postpartum woman*.* Yet other women withheld their status from partners due to prior refusal to have himself tested. *“I asked him to go and test, but he refused to go”* -Postpartum woman*.* In these cases, one could postulate that the women were trying to prevent disclosure asymmetry – not wanting to disclose when they did not know their partner’s status: *“I didn’t tell him immediately, I brought him here for his own test also, so we found out together that we were both positive” -*Postpartum woman. However, reasons for non-disclosure to family were not due to fear, but more to protect family from stress or HIV-related stigma. “*I did not disclose my status to my mother because she is sick and I knew if I told her she would be thinking as if I will die tomorrow”* -ANC woman, partner HIV-negative*.*

Women who did disclose to male partners did so mainly to maintain honest communication, they did not trust anyone else, and/or wanted to motivate their partner to test for HIV. *“I told him because I cannot live a life of secrecy*” -ANC woman, partner HIV-negative. *“There is no one I can inform about my status except my husband”* -Newly-diagnosed woman. “*I decided to tell him so that he could get tested, so we will know if we are both infected*” -Postpartum woman, partner HIV-negative. *“I told my husband and sister because I encouraged them to go and know their status*” -ANC woman, partner HIV-negative.

#### The strategic role and influence of healthcare workers in disclosure to male partners

For women who found it especially difficult to disclose to male partners, healthcare workers played a key role in facilitating disclosure and in convincing male partners to test for HIV. “*The day I was told my diagnosis, the nurse asked if she could help me disclose it to my husband, then I said she should go ahead. He was briefed about my HIV diagnosis, and then I spoke to him and asked him to come for the same HIV test too. He didn’t refuse it. He was tested too and found out he is HIV-positive*” –Newly-diagnosed woman. “*I didn’t tell him, it was the nurses that told him because I told them that if I should disclose it to him, he may not handle the issue well and I may lose my marriage, so they called him and disclosed everything to him”-*Postpartum woman. *“Initially the matron asked me if I would tell him myself and I said yes, but I couldn’t do it. She then asked me to tell him to come to clinic. He met with the matron, they discussed my result, he took it in good faith and he was also advised to go for his own test” -*Newly-diagnosed woman.

In some cases, the health care worker was a co-strategist in the disclosure process: “*You know when I came here, the nurses spoke to me and tried to explain that it is important we let our partners know about it. So I told him, when I went for antenatal the previous day, that I saw a woman crying because they told her she had HIV. So he said, ‘How would she be crying?’ So that now gave me the courage to open up...you don’t just say it outright because it is awkward. So I was just looking for a way”* -ANC woman*.*

#### Non-disclosure to female partners among HIV-positive male partners

Non-disclosure also seemed to cut both ways, as some participants reported that their male partners were already diagnosed HIV-positive and on treatment but did not disclose to their female partners until prompted by a sentinel event: *“I called him when they told me I was positive. So he came here* [clinic] *and he told us that he is already positive too, and he has been receiving his medication.”-*ANC woman*.* “*He was the first to be diagnosed; he did not tell me. He had tuberculosis, so we went to the hospital where he tested HIV positive. But when we came out of the hospital, instead of him to tell me, he did not. I confronted him and asked him why he did not tell me when he was diagnosed with HIV.”* -LTFU woman*.* In some cases women disclosed to their male partners without asking them to disclose theirs: “*I don’t know his status. He didn’t disclose it to me, but he knows my status. I wasn’t bothered about asking him. I know he did it* [HIV test]*.”* -Newly-diagnosed woman. *“I just told him. He told me it is not something we should discuss at that moment. But I don’t know his status”-*Newly-diagnosed woman*.*

#### Male partner reaction to disclosure

For majority of the women who disclosed to male partners, partner reactions were often more positive and supportive than expected– with the men continuing to provide emotional, material and financial support, despite the initial shock from disclosure. “*I thought he would take it harsh on me. But he has been very caring since I told him that I’m positive and he reminds me when it is time to take my drugs*” -ANC woman, partner HIV-negative. *“From that day* [of disclosure] *he started loving me better. But he asked me not to talk about HIV anymore since we love each other. And he still supports me” -*Postpartum woman, partner HIV-negative. “*When I went home with the news that day, I was so disturbed. So he noticed and tried to find out what was wrong with me. I couldn’t really pronounce it… He perceived that something was wrong, and he knew that I went to the hospital for some tests. So he asked if I was confirmed positive, and I said yes. He told me not to feel anxious about it, not to cry and that I should go and take the drugs.” ANC woman, partner HIV-negative.*

Very few women who disclosed to male partners reported experiencing the negative consequences feared by many women in the first place, including neglect and separation that could potentially impact on PMTCT outcomes: *“I was sick so he brought me to the clinic. That’s how he knew my status. So as we were going home he told me he could not live with me anymore. He then sent me away to my father’s house. I was there for four months. He came back six days after I delivered…He refused to give me money for transport to come for my drugs here [clinic]… That’s why I was not taking the drugs”* -LTFU woman, partner HIV-negative*.* “*Since I disclosed to him, he only gives me some money for food and even if I ask him for any other thing, he won’t listen. Before now, he wasn’t behaving like this” -*LTFU woman, partner’s status unknown.

## Discussion

Among our study population of women living with HIV in rural North-Central Nigeria, we found overall disclosure rates to anyone to be relatively high, at 81%. Male partner disclosure reported by our study participants was also relatively high at 85%, compared to 23.0%, 86.5% and 90.4% reported in previous studies among women in South-West Nigeria [9, 24, 27]. Across similar African settings, male partner disclosure rates among HIV-positive women ranged between 44% in Kenya to 93% in Zimbabwe [7, 40]. Similar to our findings, a larger proportion of African women living with HIV ultimately disclose to their male partners than to others [7, 15, 21, 41], although our findings suggest, similar to other studies [21], that family members were often the first to be disclosed to.

Fear of divorce, interpersonal/domestic violence, neglect or other forms of psychological abuse deterred women from immediately disclosing to their male partners. However, with time and encouragement, especially from healthcare workers, women in our study population disclosed with surprisingly, largely positive results, as reported elsewhere in Africa [18, 22], even in situations where male partners were reportedly HIV-negative. Among our study cohort, reasons for male partner disclosure included feelings of obligation and to encourage partner HIV testing, as reported from studies in other African countries [15, 28]. For some women, disclosure to anyone occurred on the same day or shortly after diagnosis, as reported in other Nigerian [42] and African studies [18, 29]. Similar to findings in other studies [6, 21, 43], women who did not disclose a positive HIV status to family members were seeking to protect them.

In our study, newly-diagnosed women had significantly lower disclosure rates to anyone or family, compared to women in the other cascade groups who had previously established care (*p* = 0.001). Newly-diagnosed women also had the lowest rate of knowledge of partner serostatus (*p* = 0.004). This is understandable considering that these women were newly-diagnosed and may not have had enough time to process and share their diagnosis with anyone, or seek to know partner’s HIV status. This finding is similar to disclosure data for the ART cascade that shows that newly-diagnosed patients had significantly lower disclosure rates than those in established care [5]. Postpartum and breastfeeding women in care, on the other hand, had the lowest disclosure rates to male partners. The reason for this is not clear from our study. However, Brou et al. [12] suggest that breastfeeding status correlates with partner disclosure by HIV-positive women; with women who choose exclusive formula feeding disclosing at a higher rate than those who choose to breastfeed. We were not able to explore disclosure rates in the context of infant feeding practices, as our study objectives did not include in-depth evaluation of infant feeding practices across all four cascade groups.

Pregnant women (newly diagnosed + ANC) had significantly lower disclosure to anyone than non-pregnant women (postpartum + LTFU) (*p* = 0.0007). Again, one likely explanation could be that non-pregnant women may have known their status for a longer duration. However, we were not able to accurately establish when previously-diagnosed women were diagnosed, as many women in our small rural community study setting did not enrol at the facility where they were first HIV-diagnosed: They often presented at multiple other facilities as “testing-naive” patients. Additionally, when asked, they were often unsure of the exact day or month of testing. This phenomenon was noted in the MoMent prospective study as well [44]. We are therefore limited in explaining if, or how time of HIV testing, and infant feeding practice influenced disclosure across the PMTCT cascade. Further research is needed on these aspects. There were no observed differences across the cascade groups with respect to male partner or family disclosure. However, our small sample size may have precluded the discovery of potential differences in our quantitative results.

Addressing the lack of, or delayed disclosure among couples is important for our study population and the larger HIV community in our study setting because of the relatively high reported HIV serodiscordance rate of nearly 56%, and unknown partner HIV status of 33%. Among our cascade groups, newly-diagnosed and ANC women were less likely to know their partner's status, compared to postpartum and LTFU women, and this difference was statistically significant. Previous Nigerian studies reported a similar serodiscordance rate for North-Central Nigeria of 51.9% [23] and lower rate of 38.5% for South-East Nigeria [45]. Proportions of HIV-positive women with unknown male partner status of 62.4% and 85% have been reported from studies in North-Central and South-East Nigeria, respectively [24, 45], which are much higher than for our study. Studies in similar sub-Saharan African settings have reported serodiscordance rates between 22.9% and 39% [15, 46, 47], and unknown male partner status between 32.7% and 80% among women living with HIV [11, 15, 48, 49]. Since we could not establish time of HIV diagnosis for our cascade groups, it is not possible to determine how and if this played a role in the observed differences in serodiscordance, and knowledge of partner status. For serodiscordant couples, early partner testing, notification and treatment can avert seroconversion in the HIV-negative partner [23].

Similar to previous findings [15, 43] our study highlights that healthcare workers play key motivating and supporting roles in disclosure among women living with HIV, especially to male partners, and actively facilitate partner HIV testing. Pre-existing and longstanding gender inequities in our study communities and similar settings have necessitated women needing more support (including to overcome fear) in order to disclose HIV-positive status to male partners. Intimate partner violence, inequitable laws and harmful traditional practices, including limited decision-making for women, reinforce unequal power dynamics between men and women [50]. Healthcare workers can, and have mediated these power dynamics by increasing their involvement in disclosure, especially by women to male partners. By supporting couples counselling and education on testing and treatment, healthcare workers play a crucial authoritative role in minimizing negative partner reactions for women who have accepted testing and tested positive. This is especially important, as studies show that men living with HIV whose wives know their seropositive status are often less likely to be violent or react negatively to news of female partner’s seropositivity; thus, stressing the need for mutual HIV testing and disclosure [20, 26]. Couple testing and disclosure will also lessen the burden on the partner, which in the PMTCT context is the woman, who would otherwise test first and/or positive [51, 52].

As much as healthcare workers in our study setting assisted in disclosure, they and mentor mothers could only provide counselling and psychosocial support: they were not trained to provide professional mental health services. As such, professional mental services were not available to our study participants, especially newly-diagnosed women. Mental health services, if available, are often very expensive and located at large and/or tertiary centers located in urban areas at great traveling distance from rural communities. Thus, the study team could not refer participants for these services. Furthermore, such mental health referrals are not included in routine PMTCT care at the study facilities.

While no respondent in our study reported experiencing physical violence from their male partner as a result of disclosure, fear of such intimate partner violence as well as emotional/financial neglect and divorce/separation were expressed by women across cascade groups as reasons for non-disclosure. Therefore, prevention and management of marital conflict and intimate partner violence in the context of HIV disclosure remain important issues to address in PMTCT programming.

Surprisingly, male partner disclosure rates were no different among LTFU women compared to other cascade groups in our study. This is contrary to previous findings where nondisclosure has been reported as a correlate of PMTCT cascade dropout among women living with HIV [14] [53]; however, the disclosure evaluated in these studies were to anyone and not specifically disaggregated for disclosure to male partners or other individuals. Larger, and more robust studies are needed to examine the relationship between male partner-specific disclosure rates among women in and out of care along the PMTCT cascade.

### Study limitations

This study was conducted in rural Nigeria with a purposive sample, therefore study findings may not be generalizable to all HIV-positive women in the study communities, nor to urban settings in Nigeria or elsewhere. There was also significant missing data for knowledge of male partner status: 19 of 100 women did not respond to this question. Analysis was therefore based on the remaining 81 who did. Furthermore, as explained earlier, we were unable to collect reliable data for all study participants on specific initial date of HIV diagnosis. Thus, we could not evaluate if timing of diagnosis was a correlate of overall disclosure rates, and rates between cascade groups. Socio-economic status and male partner socio-demographic data could also not be evaluated vis-à-vis disclosure rates, since we did not collect these data. Disclosure and male partner HIV-status was as reported by participants; it was not possible to verify this information and as such may not reflect reality. Lastly, limitations in cascade-based recruitment (especially for the two post-partum groups) in our rural study settings resulted in a relatively small sample size for each cascade group; this limited robust statistical comparisons between and within groups.

## Conclusions

With support from healthcare workers and irrespective of cascade status, male partner disclosure was ultimately achieved with largely positive results for the majority of women in our study. Thus, strategies to increase healthcare worker skills and active involvement are likely to yield high rates of successful male partner disclosure in rural communities - a strategy that is especially important where there are high rates of serodiscordance. Concurrent strategies to enable healthcare workers make successful contact with male partners, either at the facility or in the community, will also be needed to facilitate the disclosure process for women at any stage of the PMTCT cascade. Comprehensive healthcare worker-supported interventions that target male partner disclosure in particular and context-specific women’s empowerment in general are important to maximize outcomes in communities with high HIV burdens and low PMTCT performance. Lastly, while mental health and gender-based violence programs are limited in Nigeria and similar settings, the need is well-demonstrated [50], and it is important to establish these services in conjunction with PMTCT program scale-up.

## Abbreviations

ANC: Antenatal care
ART: Antiretroviral therapy
HIV: Human immunodeficiency virus
LTFU: Lost to follow-up
PMTCT: Prevention of mother-to-child transmission of HIV

## References

[1] UNAIDS (2017). UNAIDS Data 2017.

[2] CHAI Nigeria (2012). SMS printer program report.

[3] WHO. Programmatic Update. Antiretroviral treatment as prevention (TASP) of HIV and TB. Geneva: World Health Organisation; 2012.

[4] Hodgson I, Plummer ML, Konopka SN, Colvin CJ, Jonas E, Albertini J (2014). A systematic review of individual and contextual factors affecting ART initiation, adherence, and retention for HIV-infected pregnant and postpartum women. PLoS One.

[5] Ostermann J, Pence B, Whetten K, Yao J, Itemba D, Maro V (2015). HIV serostatus disclosure in the treatment cascade: evidence from northern Tanzania. AIDS Care.

[6] Winchester MS, McGrath JW, Kaawa-Mafigiri D, Namutiibwa F, Ssendegye G, Nalwoga A (2013). Early HIV disclosure and nondisclosure among men and women on antiretroviral treatment in Uganda. AIDS Care.

[7] Spangler SA, Onono M, Bukusi EA, Cohen CR, Turan JM (2014). HIV-positive status disclosure and use of essential PMTCT and maternal health services in rural Kenya. J Acquir Immune Defic Syndr.

[8] Falang KD, Akubaka P, Jimam NS (2012). Patient factors impacting antiretroviral drug adherence in a Nigerian tertiary hospital. J Pharmacol Pharmacother.

[9] Ekama SO, Herbertson EC, Addeh EJ, Gab-Okafor CV, Onwujekwe DI, Tayo F (2012). Pattern and determinants of antiretroviral drug adherence among Nigerian pregnant women. J Pregnancy.

[10] Charurat M, Oyegunle M, Benjamin R, Habib A, Eze E, Ele P (2010). Patient retention and adherence to antiretrovirals in a large antiretroviral therapy program in Nigeria: a longitudinal analysis for risk factors. PLoS One.

[11] Peltzer K, Jones D, Weiss SM, Villar-Loubet O, Shikwane E (2013). Sexual risk, serostatus and intimate partner violence among couples during pregnancy in rural South Africa. AIDS Behav.

[12] Brou H, Djohan G, Becquet R, Allou G, Ekouevi DK, Viho I (2007). When do HIV-infected women disclose their HIV status to their male partner and why? A study in a PMTCT programme, Abidjan. PLoS Med.

[13] Stringer EM, Chi BH, Chintu N, Creek TL, Ekouevi DK, Coetzee D (2008). Monitoring effectiveness of programmes to prevent mother-to-child HIV transmission in lower-income countries. Bull World Health Organ.

[14] Woldesenbet S, Jackson D, Lombard C, Dinh TH, Puren A, Sherman G (2015). Missed opportunities along the prevention of mother-to-child transmission services Cascade in South Africa: uptake, determinants, and attributable risk (the SAPMTCTE). PLoS One.

[15] Sendo EG, Cherie A, Erku TA (2013). Disclosure experience to partner and its effect on intention to utilize prevention of mother to child transmission service among HIV positive pregnant women attending antenatal care in Addis Ababa, Ethiopia. BMC Public Health.

[16] Tam M, Amzel A, Phelps BR (2015). Disclosure of HIV serostatus among pregnant and postpartum women in sub-Saharan Africa: a systematic review. AIDS Care.

[17] Medley A, Garcia-Moreno C, McGill S, Maman S (2004). Rates, barriers and outcomes of HIV serostatus disclosure among women in developing countries: implications for prevention of mother-to-child transmission programmes. Bull World Health Organ.

[18] Kiula ES, Damian DJ, Msuya SE (2013). Predictors of HIV serostatus disclosure to partners among HIV-positive pregnant women in Morogoro, Tanzania. BMC Public Health.

[19] Alemayehu M, Aregay A, Kalayu A, Yebyo H (2014). HIV disclosure to sexual partner and associated factors among women attending ART clinic at Mekelle hospital, northern Ethiopia. BMC Public Health.

[20] Colombini M, James C, Ndwiga C, Mayhew SH (2016). The risks of partner violence following HIV status disclosure, and health service responses: narratives of women attending reproductive health services in Kenya. J Int AIDS Soc.

[21] Visser MJ, Neufeld S, de Villiers A, Makin JD, Forsyth BW (2008). To tell or not to tell: south African women's disclosure of HIV status during pregnancy. AIDS Care.

[22] Rujumba J, Neema S, Byamugisha R, Tylleskar T, Tumwine JK, Heggenhougen HK (2012). "Telling my husband I have HIV is too heavy to come out of my mouth": pregnant women's disclosure experiences and support needs following antenatal HIV testing in eastern Uganda. J Int AIDS Soc.

[23] Onovo AA, Nta IE, Onah AA, Okolo CA, Aliyu A, Dakum P (2015). Partner HIV serostatus disclosure and determinants of serodiscordance among prevention of mother to child transmission clients in Nigeria. BMC Public Health.

[24] Adekanle DA, Olowookere SA, Adewole AD, Adeleke NA, Abioye-Kuteyi EA, Ijadunola MY (2015). Sexual experiences of married HIV positive women in Osogbo, southwest Nigeria: role of inappropriate status disclosure. BMC Womens Health.

[25] Crankshaw TL, Voce A, King RL, Giddy J, Sheon NM, Butler LM (2014). Double disclosure bind: complexities of communicating an HIV diagnosis in the context of unintended pregnancy in Durban, South Africa. AIDS Behav.

[26] Mulrenan C, Colombini M, Howard N, Kikuvi J, Mayhew SH (2015). Exploring risk of experiencing intimate partner violence after HIV infection: a qualitative study among women with HIV attending postnatal services in Swaziland. BMJ Open.

[27] Ezechi OC, Gab-Okafor C, Onwujekwe DI, Adu RA, Amadi E, Herbertson E (2009). Intimate partner violence and correlates in pregnant HIV positive Nigerians. Arch Gynecol Obstet.

[28] Maman S, van Rooyen H, Groves AK (2014). HIV status disclosure to families for social support in South Africa (NIMH project accept/HPTN 043). AIDS Care.

[29] Abdool Karim Q, Dellar RC, Bearnot B, Werner L, Frohlich JA, Kharsany AB (2015). HIV-positive status disclosure in patients in care in rural South Africa: implications for scaling up treatment and prevention interventions. AIDS Behav.

[30] Ogoina D, Ikuabe P, Ebuenyi I, Harry T, Inatimi O, Chukwueke O (2015). Types and predictors of partner reactions to HIV status disclosure among HIV-infected adult Nigerians in a tertiary hospital in the Niger Delta. Afr Health Sci.

[31] Atuyambe LM, Ssegujja E, Ssali S, Tumwine C, Nekesa N, Nannungi A (2014). HIV/AIDS status disclosure increases support, behavioural change and, HIV prevention in the long term: a case for an Urban Clinic, Kampala, Uganda. BMC Health Serv Res.

[32] Titilope AA, Adediran A, Umeh C, Akinbami A, Unigwe O, Akanmu AS (2011). Psychosocial Impact Psychosocial Impact Of disclosure of HIV Serostatus in heterosexual relationship at the Lagos University teaching hospital, Nigeria. Nigerian Med J.

[33] Ebuenyi ID, Ogoina D, Ikuabe PO, Harry TC, Inatimi O, Chukwueke OU (2014). Prevalence Pattern and Determinants of disclosure of HIV status in an anti retroviral therapy Clinic in the Niger Delta Region of Nigeria. African J Infect Dis.

[34] Adebayo AM, Ilesanmi OS, Omotoso BA, Ayodeji OO, Kareem AO, Alele FO (2014). Disclosure To sexual partner and condom use among HIV positive clients attending ART clinic at a tertiary health facility in south West Nigeria. Pan Afric Med J.

[35] International NaI (2014). Nigeria Demographic and Health Survey 2013.

[36] UNAIDS (2016). On the fast track to an AIDS-free generation: the incredible journey of the global plan towards the elimination of new HIV infections among children by 2015 and keeping their mothers alive joint.

[37] Sam-Agudu NA, Cornelius LJ, Okundaye JN, Adeyemi OA, Isah HO, Wiwa OM (2014). The impact of mentor mother programs on PMTCT service uptake and retention-in-Care at Primary Health Care Facilities in Nigeria: a prospective cohort study (MoMent Nigeria). J Acquir Immune Defic Syndr.

[38] Federal Ministry of Health Nigeria. National Guidelines for Prevention of Mother-to-Child Transmission of HIV. Abuja: Federal Ministry of Health Nigeria; 2010.

[39] Glaser BG, & Strauss, Anselm L.. The discovery of grounded theory: Strategies for qualitative research: Transaction Publishers; 2009.

[40] Shamu S, Zarowsky C, Shefer T, Temmerman M, Abrahams N (2014). Intimate partner violence after disclosure of HIV test results among pregnant women in Harare, Zimbabwe. PLoS One.

[41] Patel R, Ratner J, Gore-Felton C, Kadzirange G, Woelk G, Katzenstein D (2012). HIV disclosure patterns, predictors, and psychosocial correlates among HIV positive women in Zimbabwe. AIDS Care.

[42] Amoran OE (2012). Predictors of disclosure of sero-status to sexual partners among people living with HIV/AIDS in Ogun state, Nigeria. Niger J Clin Pract.

[43] Madiba S, Letsoalo R (2013). HIV Disclosure to partners and family among women enrolled in prevention of mother to child transmission of HIV program: implications for infant feeding in poor resourced communities in South Africa. Glob J Health Sci.

[44] Sam-Agudu NA, Ramadhani HO, Isah C, Anaba U, Erekaha S, Fan-Osuala C (2017). The impact of structured mentor mother programs on 6-month postpartum retention and viral suppression among HIV-positive women in rural Nigeria: a prospective paired cohort study. J Acquir Immune Defic Syndr.

[45] Lawani LO, Onyebuchi AK, Iyoke CA (2014). Dual Method use for protection of pregnancy and disease prevention among HIV-infected women in south East Nigeria. BMC Womens Health.

[46] Feyissa TR, Melka AS (2014). Demand for modern family planning among married women living with HIV in western Ethiopia. PLoS One.

[47] Kaida A, Matthews LT, Kanters S, Kabakyenga J, Muzoora C, Mocello AR (2013). Incidence and predictors of pregnancy among a cohort of HIV-positive women initiating antiretroviral therapy in Mbarara, Uganda. PLoS One.

[48] Matthews LT, Smit JA, Moore L, Milford C, Greener R, Mosery FN (2015). Periconception HIV risk behavior among men and women reporting HIV-Serodiscordant partners in KwaZulu-Natal, South Africa. AIDS Behav.

[49] Matthews LT, Moore L, Crankshaw TL, Milford C, Mosery FN, Greener R (2014). South Africans with recent pregnancy rarely know partner's HIV serostatus: implications for serodiscordant couples interventions. BMC Public Health.

[50] AVERT. Gender Inequality and HIV. 2017. https://www.avert.org/professionals/social-issues/gender-inequality. Accessed 27 Jan 2018.

[51] Ateka GK (2006). HIV status disclosure and partner discordance: a public health dilemma. Public Health.

[52] Roxby AC, Matemo D, Drake AL, Kinuthia J, John-Stewart GC, Ongecha-Owuor F (2013). Pregnant women and disclosure to sexual partners after testing HIV-1-seropositive during antenatal care. AIDS Patient Care STDs.

[53] Watson-Jones D, Balira R, Ross DA, Weiss HA, Mabey D (2012). Missed opportunities: poor linkage into ongoing care for HIV-positive pregnant women in Mwanza, Tanzania. PLoS One.

